# Beyond One Health—Zoological Medicine in the Anthropocene

**DOI:** 10.3389/fvets.2017.00102

**Published:** 2017-06-29

**Authors:** Chris Walzer

**Affiliations:** ^1^Department of Integrative Biology and Evolution, University of Veterinary Medicine Vienna, Vienna, Austria

**Keywords:** zoological medicine, zoo veterinarian, wildlife, anthropogenic pressures, One Health

In contrast to some of the well-established core disciplines of veterinary medicine, such as radiology, surgery, and internal medicine, zoological medicine is often perceived as a relatively recent development. However, as early as 1831, local veterinary practitioner Charles Spooner became the first zoo veterinarian at the London Zoological Garden in the United Kingdom. Shortly thereafter, he was followed by William Youatt, who remained in that position for 17 years while also establishing the world’s first veterinary journal, the *Veterinarian*, which reported on the diseases of wild animals. In 1865, the zoo also hired a pathologist. During the same period, in 1870, Max Schmidt, the director of the Zoological Garden in Frankfurt am Main in Germany, wrote *Vergleichende Pathologie und Pathologische Anatomie der Säugetiere und Vögel* (Comparative Pathology and Pathological Anatomy of mammals and Birds) (1). In North America, the Philadelphia Zoo employed a pathologist in 1901, and in the same year the New York Zoological Society (now the Wildlife Conservation Society) established the first zoological medical department with Frank H. Miller as veterinarian and Harlow Brooks as pathologist (1).

It was in 1946 that a small group of zoo veterinarians convened at the annual American Veterinary Medical Association (AVMA) meeting in Boston to form the Zoo Veterinarians group from which the present-day American Association of Zoo Veterinarians (AAZV) emerged in 1968. From 1970 onward, the AAZV published the *Journal of Zoo Animal Medicine* (changed to the *Journal of Zoo and Wildlife Medicine* in 1989). A few years after the establishment of the Zoo Veterinarians group, in 1951, a group of US and Canadian wildlife biologists founded an organization called the Wildlife Disease Committee. The committee shortly thereafter morphed into the Wildlife Disease Association, establishing an international scientific organization dedicated to the study of wild animal health. In 1965, the organization’s newsletter grew into a journal entitled the *Bulletin of the Wildlife Disease Association*, which was later expanded to become the *Journal of Wildlife Diseases*. During the same time, the International Association for Aquatic Animal Medicine, an organization of individuals who professionally practice aquatic animal medicine, teach, and conduct research in aquatic animal medicine, was created. Shortly thereafter in 1978, the American Association of Wildlife Veterinarians was established.

In Europe, Rudolf Ippen established yearly International Symposia on the Diseases of Zoo and Wild Animals in 1959. Together with the Research Station for Vertebrate Research (now Leibniz Institute of Zoo and Wildlife Research, IZW), established in 1973 by the Academy of Sciences in the former German Democratic Republic, these annual conferences firmly established and drove zoological medicine forward. In 1993, symposium participants established the European Association of Zoo and Wildlife Veterinarians. The AVMA–American Board of Veterinary Specialists recognized zoological medicine (ACZM) as a speciality in 1988, and similarly the European College of Zoological Medicine was established and enlarged between 1993 and 2012. Several further specialist organizations were formed over the years, among others, the Association of Avian Veterinarians in 1980 and 10 years later the Association of Reptilian and Amphibian Veterinarians. In addition, the past decades have seen numerous regional and national associations dealing with wildlife health arising worldwide.

Since the first zoo veterinarian was employed 1831 in London, the world’s human population has grown from a mere 1 billion to over 7.5 billion people in 2017. Today, practically all species live in dynamic multiuse landscapes in which anthropogenic activities have become the main driver of change. We have clearly left the unusually stable 10,000 years of the Holocene behind us and have firmly entered into the Anthropocene (2). Due largely to the reliance on fossil fuels, expansion of industrial agriculture and land transformation in a globalized society dominated by deregulated markets that rely on constant growth, the earth’s regulatory mechanisms, which in the past provided regular temperatures, freshwater availability, and guaranteed biogeochemical flows, are being severely perturbed. Humankind is potentially irreversibly leaving the safe operating space for human existence on Earth (3). In addition, global sociopolitical turmoil, with starkly increasing economic inequality, polarized value systems, rejection of science, and subsequent disregard for evidence-based policy decisions, asymmetrically shifts cost–benefit ratios away from sustainable environmental and biodiversity conservation efforts toward further unbridled economic development. It is within this context that I discuss the opportunities, challenges, and limitations that zoological medicine faces today when working with wildlife.

At the outset, it is important to realize that, while scientific communities consistently strive forwards in ever-narrowing, clearly delineated, and possibly protectionist speciality fields reflected in our respective organizations, specialization degrees, and sectoral resource allocation, in actuality there is but one “wildlife.” This holds true irrespective of animals’ housing conditions and context. Today, all wildlife survives along a gradient of increasing human encroachment and disturbance, from the habituated pet green iguana (*Iguana iguana*) *via* the zoo-housed and behaviorally conditioned African elephant (*Loxodonta africana*) to the free-ranging Asiatic wild ass (*Equus hemionus*) in the Mongolian Gobi. While, in the past, living conditions were seen as vastly different between zoo-housed and free-ranging individuals, these borders have become more permeable in the past decade. As anthropogenic encroachment continues, free-ranging wildlife subsists on ever-smaller islands, often fenced and frighteningly similar to zoos, in an ocean of human development.

Not only has our environment changed in the past decades but so has our understanding of health and disease. Wildlife health can no longer be viewed as simply the absence of disease, both disease and health being products of complex interactions and systems. Unfortunately, definition of wildlife health along the proposed gradient appears in many instances entrenched in and focused on disease (4).

In this challenging environment, seemingly novel cross-disciplinary approaches to human, animal, and environmental health have been developed, defined, and branded. One Health, Conservation Medicine, and Ecosystem Health are but a few of the concepts that are being widely discussed and equally hyped. The One Health paradigm recognizes that human and animal health and their respective environments are inextricably linked (5). While the One Health concept is often touted as a novel approach, the impact of environmental factors on human health can actually be traced back to Hippocrates (c.460 BCE–c.370 BCE). One Health approaches are frequently anthropocentric and human medical driven, which in the past two decades has exacerbated the parasite–pathogen-focused perspective of wildlife health by primarily highlighting wildlife as a source of illness, emerging infectious diseases, and threats to public health while neglecting the value of biodiversity and the associated services (4, 6, 7).

Today consensus exists that wildlife health, like human health, must be viewed beyond parasites and pathogens, incorporating social evolutionary and environmental factors while considering individual attributes and behaviors (4). In order for veterinarians to fully participate and even lead in the field of wildlife health in the future, they must necessarily embrace a holistic approach to health and be fully aware of and understand the pressing present-day conservation challenges (6). Wildlife health incorporates the capacity to cope with change and results from complex dynamic interactions of biologic, environmental, and socioeconomic factors (4). Clearly, a modern and all-inclusive approach to zoological medicine must integrate input across previously distinct disciplines such as physiology, ecology, animal behavior, conservation biology, economics, social sciences, and many more. Recently, a publication-based survey showed that, while the field of One Health was growing rapidly (nearly 15% increase of publications per year), its impacts still clustered into three distinct communities: ecologists, veterinarians, and a third community consisting of population biologists, mathematicians, epidemiologists, and experts in human health. Particularly worrying was the obstinate persistence of the traditional veterinary medicine and ecology silos (8). Integrating fitness, life-history traits, and trade-offs when evaluating wildlife health in the face of chronic stress from anthropogenic pressures appears indispensable.

While intensive work within a discipline is essential when developing expertise, it is equally clear that solving problems that impact the continued development of human societies, including the maintenance of wildlife health and the provision of ecosystem services, the benefits people obtain from ecosystems that are the basis for all life, necessitates research and practice that bridges the traditional disciplinary silos. The present-day gaps in knowledge regarding sustainable health management at the animal–human–ecosystem interfaces highlight the fact that these issues involve highly dynamic and interconnected rather than reductionist and straightforward processes. It appears essential to reconcile the evolving and complex nature of these issues with specific and appropriate problem solving approaches. In contrast, solutions based on naïve simplification of interdependencies or complex dynamic infectious disease models with inadequate ground truthing leads to results that are ultimately not relevant when informing policy and implementing management. Furthermore, experience in the past 25 years indicates that health issues at the various interfaces and global biodiversity conservation in general are most likely so-called “wicked or even super wicked problems” implying the need for novel approaches when addressing these issues (9). Based on these experiences, it seems clear that the usual backward looking method of investigating the past and generating selective and singular predictions is only sufficient for “tame problems” but wholly inadequate for the present-day highly dynamic and interconnected environmental conservation and wildlife health issues. It is essential to apply a forward reasoning approach that identifies possible future scenarios while integrating uncertainties.

Overcoming the traditional disciplinary boundaries while integrating novel approaches in describing and evaluating health in an ever-changing environment constitutes the major challenge to zoological medicine in the future. It has been pointed out that, among others, quantitative Bayesian modeling approaches offer opportunities to integrate data from veterinary medicine with ecological concepts and mathematical epidemiology (8). Access to, novel technologies such as portable real-time PCRs and genome sequencing, eDNA sampling for pathogens, and a multitude of functional immunological tests further facilitate integration across disciplines (10). Beyond generating robust data, translating zoological medical science outputs to inform policy and drive action is a demanding imperative that is in dire need of future cross- disciplinary participatory approaches.

The zoological medicine section strives to publish high-level basic and clinical research that furthers the knowledge and understanding of health and disease in the broadest multidisciplinary sense of the term along a gradient from captive to free-ranging wildlife and across all taxa. With this understanding, the section encourages multidisciplinary and interdisciplinary submissions that recognize that wildlife health and resilience are the result of interactions between socioeconomic and environmental factors, as well as the traits of individuals and populations. Here, the transdisciplinary, ecology-driven, and conservation-centered Conservation Medicine approach can provide guidance (6, 11). In accordance with the One Health Initiative, submissions related to the human–domestic pet–livestock–wildlife interface are encouraged. The scope of this section includes not only the core fields of veterinary medicine such as surgery, anesthesia, physiology, pathology, immunology, anatomy, epidemiology, and animal welfare as they relate to wildlife but clearly also incorporates fields including but not limited to ecology, conservation biology, economics, and the social sciences. The development of innovative techniques and equipment for the diagnosis, and where appropriate, the treatment of wildlife disease are equally welcome. Case reports and disease outbreaks descriptions that present novel insights into wildlife health will be similarly considered.

## Author Contributions

The author confirms being the sole contributor of this work and approved it for publication.

## Conflict of Interest Statement

The author declares that the research was conducted in the absence of any commercial or financial relationships that could be construed as a potential conflict of interest.

## References

[1] Cook RA Miller RE Veterinary medicine. In: Bell CE, editor. Encyclopedia of the World’s Zoos. Chicago, IL: Fitzroy Dearborn Publishers (2001). p. 1291–7.

[2] Rockström J Steffen W Noone K Persson A Chapin FS III Lambin EF A safe operating space for humanity. Nature (2009) 461(7263):472–5. 10.1038/461472a 19779433

[3] Steffen W Richardson K Rockstrom J Cornell SE Fetzer I Bennett EM Planetary boundaries: guiding human development on a changing planet. Science (2015) 347(6223):1259855 10.1126/science.1259855 25592418

[4] Stephen C. Toward a modernized definition of wildlife health. J Wildl Dis (2014) 50(3):427–30. 10.7589/2013-11-305 24807179

[5] Evans BR Leighton FA. A history of one health. Rev Sci Tech (2014) 33(2):413–20. 10.20506/rst.33.2.2298 25707172

[6] Deem SL Conservation medicine to one health: the role of zoologic veterinarians. In: Miller RE Fowler M, editors. Fowler’s Zoo and Wild Animal Medicine. St. Louis, MO: Saunders (2015). p. 698–703.

[7] Rüegg SR McMahon BJ Häsler B Esposito R Nielsen LR Ifejika Speranza C A blueprint to evaluate one health. Front Public Health (2017) 5:20. 10.3389/fpubh.2017.00020 28261580 PMC5311072

[8] Manlove KR Walker JG Craft ME Huyvaert KP Joseph MB Miller RS “One health” or three? Publication silos among the one health disciplines. PLoS Biol (2016) 14(4):e1002448 10.1371/journal.pbio.1002448 27100532 PMC4839662

[9] Rittel H Webber M Dilemmas in a general theory of planning. Policy Sci (1973) 4(2):155–69. 10.1007/BF01405730

[10] Marx V. PCR heads into the field. Nat Methods (2015) 12(5):393–7. 10.1038/nmeth.3369 25924072

[11] Tabor GM Defining conservation medicine. In: Aguirre AA Ostfeld RS Tabor G House C Pearl MC, editors. Conservation Medicine Ecological Health in Practice. New York: Oxford University Press (2002). p. 8–16.

